# Evaluation of potential molecular interaction between quorum sensing receptor, LuxP and grouper fatty acids: in-silico screening and simulation

**DOI:** 10.7717/peerj.6568

**Published:** 2019-04-05

**Authors:** Chen-Fei Low, Mohd Shahir Shamsir, Zeti-Azura Mohamed-Hussein, Syarul Nataqain Baharum

**Affiliations:** 1 Institute of Systems Biology (INBIOSIS), Universiti Kebangsaan Malaysia, Bangi, Selangor, Malaysia; 2 Faculty of Bioscience and Bioengineering, Universiti Teknologi Malaysia, Skudai, Johor, Malaysia; 3 Centre for Bioinformatics Research, Institute of Systems Biology (INBIOSIS), Universiti Kebangsaan Malaysia, Bangi, Selangor, Malaysia; 4 Centre for Frontier Sciences, Faculty of Science and Technology, Universiti Kebangsaan Malaysia, Bangi, Selangor, Malaysia

**Keywords:** AI-2 quorum sensing, Grouper metabolites, Fatty acids, Molecular dynamic analysis, Molecular interaction

## Abstract

Pathologically relevant behaviors of *Vibrio*, such as the expression of virulence factors, biofilm production, and swarming motility, have been shown to be controlled by quorum sensing. The autoinducer-2 quorum sensing receptor protein LuxP is one of the target proteins for drug development to suppress the virulence of *Vibrio*. Here, we reported the potential molecular interaction of fatty acids identified in vibriosis-resistant grouper with LuxP. Fatty acid, 4-oxodocosahexaenoic acid (4R8) showed significant binding affinity toward LuxP (−6.0 kcal/mol) based on molecular docking analysis. The dynamic behavior of the protein–ligand complex was illustrated by molecular dynamic simulations. The fluctuation of the protein backbone, the stability of ligand binding, and hydrogen bond interactions were assessed, suggesting 4R8 possesses potential interaction with LuxP, which was supported by the low binding free energy (−29.144 kJ/mol) calculated using the molecular mechanics Poisson–Boltzmann surface area.

## Introduction

The aquaculture of brown-marbled grouper suffers from a high frequency of vibriosis outbreak, which often causes massive mortality. Thus, vibriosis-resistant grouper is of great interest to the aquaculture industry, as it could reduce economic losses and facilitate aquaculture management. Studies have been conducted to comprehend the disease etiology, and a marker-assisted selective breeding scheme has been developed to reproduce grouper offspring with greater disease resistance. Disease resistance in grouper has been extensively studied at the molecular level through transcriptomics (Huang et al., 2011; Low et al., 2014a, 2015a; Mu et al., 2010), proteomics (Low et al., 2014b, 2015b), and metabolomics approaches (Johnson & Brown, 2011; Karakach et al., 2009). In addition to extensive studies of several metabolites with antibacterial properties (Dee & Gradle, 2011; Desbois & Smith, 2010; Heath & Rock, 2004; Ouattara et al., 1997; Zheng et al., 2005), a recently conducted study has identified highly abundant metabolites, such as icosapentaenoic acid, eicosa-8,11,14-trienoic acid, and linoleic acid in brown-marbled grouper, *Epinephelus fuscoguttatus,* which has resisted *Vibrio vulnificus* infection (Nurdalila, Mayalvanan & Baharum, 2019). Study by Zhao et al. (2015) on the global metabolic response of tilapia against streptococcosis showed the involvement of specific metabolites in fish defense system against bacterial infection where they have identified l-proline contributes to the increased survival rate. While study on the *V. vulnificus* resistance in grouper has identified several fatty acids that were highly abundant during infection (Nurdalila, Mayalvanan & Baharum, 2019), nine fatty acids were selected to evaluate their potential molecular interaction with quorum sensing receptor, LuxP through molecular docking and simulation analysis. The findings from this experiment would support the importance of specific metabolites in fish defense against bacterial infection.

Quorum sensing is a bacterial cell-to-cell communication that allows a population of pathogenic bacteria to coordinate their gene expression, achieving collective behavior to evade the host immune system, to express toxic virulence factors and form antibiotic-resistant biofilms (Annous, Fratamico & Smith, 2009; Kim, Lee & Choi, 2012; Kim et al., 2003; Liang et al., 2007; Liu et al., 2013). Quorum sensing system regulation in *V. harveyi* is well characterized (Chen et al., 2002; Liu et al., 2013; Zhu et al., 2012). In *V. harveyi*, the quorum sensing system is activated by a boron-containing signaling molecule, furanosyl borate diester, that binds to the periplasmic receptor protein LuxP (Chen et al., 2002), which then forms a complex with LuxQ, a membrane protein. The activated LuxPQ complex dephosphorylates the downstream proteins LuxU and LuxO and subsequently activates the transcription of the luciferase targeted genes (Liu et al., 2013; Schauder et al., 2001; Zhu et al., 2002, 2012), leading to the expression of bioluminescence, biofilm formation and siderophore and metalloprotease production (Guo et al., 2013). In the attempt to suppress the expression of the virulence genes regulated by the quorum sensing system (Amara et al., 2010; Hentzer et al., 2002; Kalia, 2013; Guo et al., 2013; Rasmussen & Givskov, 2006; Saedi et al., 2012), studies have identified a range of secondary metabolites from microorganism (Morohoshi et al., 2008; Park et al., 2005; Rasmussen et al., 2005; Swem et al., 2009; Teasdale et al., 2009) and plant species (Packiavathy et al., 2012) that possess potent inhibitory properties against quorum sensing. In addition, it has recently been reviewed that fatty acid, cis-2-decenoic acid possesses inhibitory properties against biofilm production that was regulated by quorum sensing (Marques, Davies & Sauer, 2015). Antibiofilm activity has also been demonstrated by mono-unsaturated chain fatty acids, palmitoleic, and myristoleic acids (Nicol et al., 2018). Even though mammalian enzymes that hydrolyze the quorum sensing signaling molecules have been identified and characterized, but these enzymes were reported to be absent in model fish species (Morohoshi et al., 2008; Yang et al., 2005). Therefore, it was proposed that fish species might possess metabolic strategies to suppress bacterial pathogenicity and prevent infections (Nurdalila, Mayalvanan & Baharum, 2019; (Zhao et al., 2014, 2015). The potential of previously identified fatty acids (Nurdalila, Mayalvanan & Baharum, 2019) to interact with autoinducer-2 (AI-2) quorum sensing receptor were assessed by docking and molecular dynamic simulations in this study. Through this computational approach, the list of fatty acids was screened by AutoDockVina (Trott & Olson, 2010) to select for the best docking position with the lowest binding affinity score toward LuxP receptor protein. The selected protein–ligand complexes were then examined by molecular dynamic simulations using GROMACS (Van der Spoel et al., 2013; Hess et al., 2008) to evaluate the stability of the structure.

## Materials and Methods

### Computational methods

#### Preparation of protein receptors, ligands, and reference compounds

Autoinducer 2-binding periplasmic LuxP protein sequence of *V. vulnificus* was obtained from the NCBI Protein Databank (accession number: WP_011152474). I-TASSER (http://zhanglab.ccmb.med.umich.edu/I-TASSER/) was used to predict the 3D structure of this protein. The top-ranked model was selected for further analysis and named as *V. vulnificus* LuxP (*vv*LuxP) throughout this paper. The crystallographic co-ordinates for LuxP *V. harveyi* (PDB ID: 1JX6), named as *V. harveyi* LuxP (*vh*LuxP) throughout this paper, were selected and included in the study to serve as a control model for *vv*LuxP. The model was prepared by removing the endogenous ligand (furanosyl borate diester) and water molecules using AutoDockTools-1.5.6, and the hydrogen atoms were added to the structure. The fatty acid structure coordinates were obtained from Ligand Expo in the Protein Data Bank (http://ligand-expo.rcsb.org/). Three-dimensional structures of the reference compounds were generated using an online open access tool (https://web.chemdoodle.com). These reference compounds were randomly selected based on the findings that they can inhibit quorum sensing in *V. harveyi* (Zhu et al., 2012). Molecules were converted into the PDBQT file format prior to molecular docking.

#### Docking and molecular dynamic simulations of protein–ligand complexes

A grid box of size 30 × 30 × 30 A^3^ was generated to contain the LuxP protein, with the LuxP binding pocket set as a centroid using AutoDockTools-1.5.6. Fatty acids and reference compounds were docked in LuxP receptor binding pocket using AutoDockVina 1.1.2 (Trott & Olson, 2010). The fatty acids with closest approximate affinity toward LuxP receptor compared to the reference compounds were shortlisted and further analyzed by molecular dynamic simulations using GROMACS 4.6.5 (Van der Spoel et al., 2013; Hess et al., 2008). Protonation and structure minimization was performed using the GROMOS 54A7 force field, where hydrogens were added for optimal hydrogen bond network by default. Topology files for molecules were generated using PRODRG server (http://davapc1.bioch.dundee.ac.uk/cgi-bin/prodrg). LuxP–ligand complexes were solvated and fully immersed in the center of a cubic box prior to electrostatic energy calculation. A default 3-point model of SPC water model of GROMACS was used to solvate the box. The number of water molecules adopted to solvate the complex were as follow: vhLuxP_4R8 (23,331 molecules); vhLuxP_EPA (23,333 molecules); vhLuxP_Lax (23,331 molecules); vhLuxP_C19 (23,336 molecules); vhLuxP_C31 (23,338 molecules); vvLuxP_4R8 (22,425 molecules); vvLuxP_EPA (22,427 molecules); vvLuxP_LAX (22,430 molecules); vvLuxP_LAX#2 (22,417 molecules); vvLuxP_C19 (22,429 molecules); and vvLuxP_C31 (22,428 molecules). Electrostatic energy was calculated using gromacs preprocessor, and the system was neutralized by adding in accordance Na^+^ ions or Cl^−^ ions to create zero charged system, and subsequent energy minimization was performed. The energy minimization was performed using the steepest descent minimization of 5,000 steps (maximum number of minimization steps to perform). Energy minimization was stopped when the maximum force was less than 1.0 kJ/mol. The system was further equilibrated for 50 ps at constant volume and a temperature of 293 K. The molecular dynamic simulations were run for 10,000 ps for each protein–ligand complex, where the coordinates were saved every two ps interval. LINear Constraint Solver, LINCS algorithm was applied to constraint all bonds, including heavy atom-H bonds during the molecular dynamics (MD) simulations. Long-range electrostatic interactions were treated with the adoption of Particle Mesh Ewald method (Chen, Wang & Zhu, 2014), and the cut-off distances for the long range electrostatic and Van der Waals interactions were set at 1.0 nm. Lastly, the trajectories were saved for further analysis using the Xmgrace and UCSF Chimera (Pettersen et al., 2004).

#### Re-scoring of protein–ligand complexes using interaction energy and MM-PBSA approach

The interaction energy of the protein–ligand complexes was calculated using molecular mechanics Poisson–Boltzmann surface area (MMPBSA) (Kumari et al., 2014) tool in Gromacs. The binding energy components were calculated separately as the MM, PB, and SA energy. The binding free energy of each complex was calculated from 20 snapshots at time intervals of 0.5 from the 10 ns MD production run. In general, the binding free energy (Δ*G*_bind_) of fatty acids/reference compounds at LuxP receptor protein was calculated as follows:

(1) $${G_{{\rm{bind}}}} = {G_{{\rm{complex}}}}-{G_{{\rm{protein}}}}-{G_{{\rm{ligand}}}}$$

where *G*_complex_, *G*_protein_, *G*_ligand_ are the free energies of the complex, LuxP receptor protein, and fatty acid/reference compound, respectively. The free energy (*G*) of each state was calculated as follows:

(2) $$G = {E_{{\rm{MM}}}} + {G_{{\rm{PB}}}} + {G_{{\rm{SA}}}}-{\rm{TS}}$$

(3) $${E_{{\rm{MM}}}} = {E_{{\rm{vdw}}}} + {E_{{\rm{ele}}}} + {E_{{\rm{int}}}}$$

where *E*_MM_ is the molecular mechanical energy, *G*_PB_ and *G*_SA_ are the polar and nonpolar terms of the free energy, and TS is the entropic contribution of the solute. The solvent accessible surface area (SASA) and solvent accessible volume (SAV) models were used to calculate its contribution to the binding free energy of the complexes.

## Results

### Comparative modeling of LuxP protein

**Figure 1:**
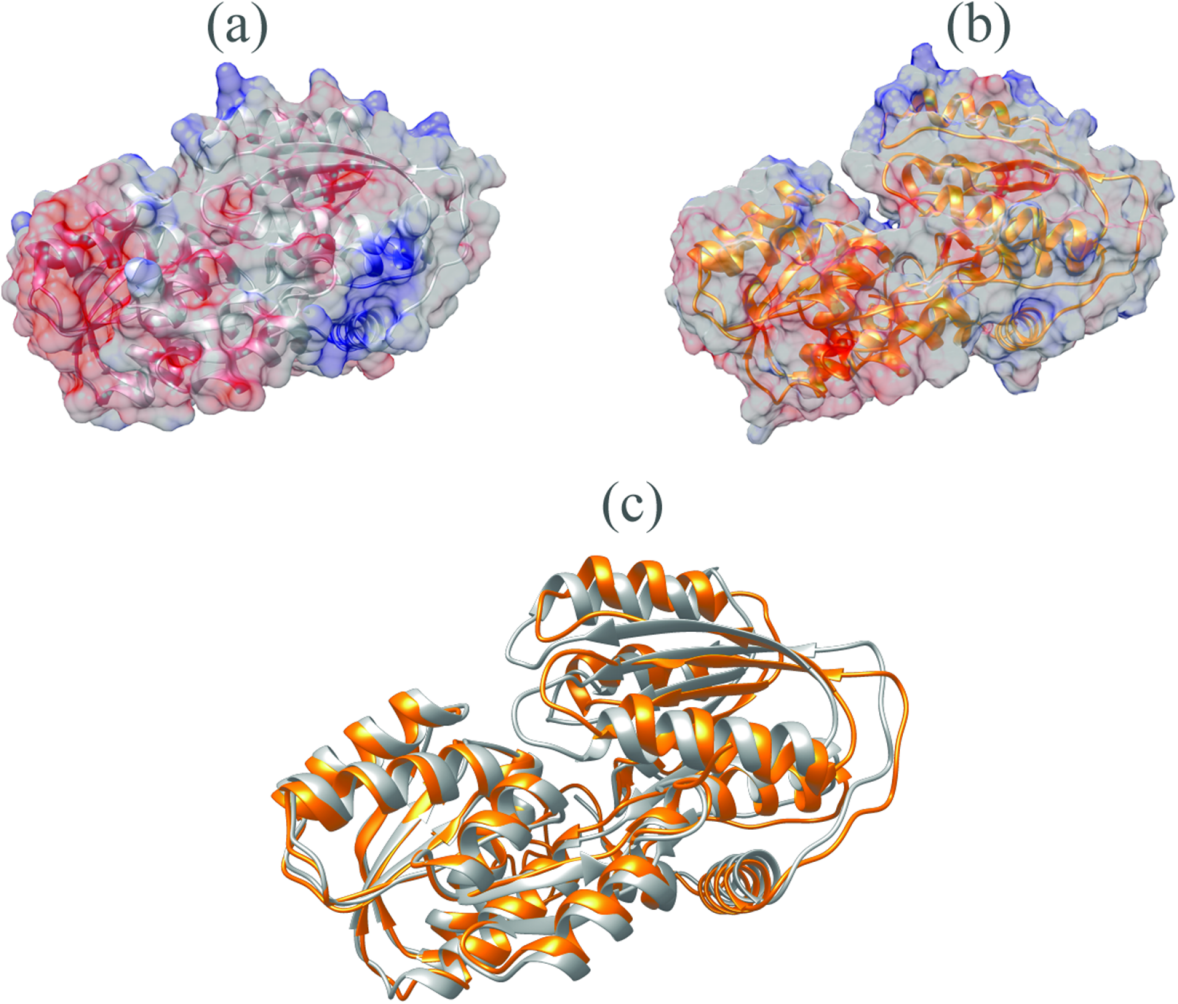
Surface electrostatic potential of crystal (A) and homology model (B) calculated according to Coulomb’s law. Homology model presents an accessible open binding pocket compared to the holo-structure of crystallized LuxP (A). Superimposition of the crystal and homology model (C) shows protein folding in loops, helices and beta-sheets that contribute to the conformational changes upon ligand binding in LuxP. Grey chain: crystal model of LuxP; orange chain: homology model.

**Figure 2:**
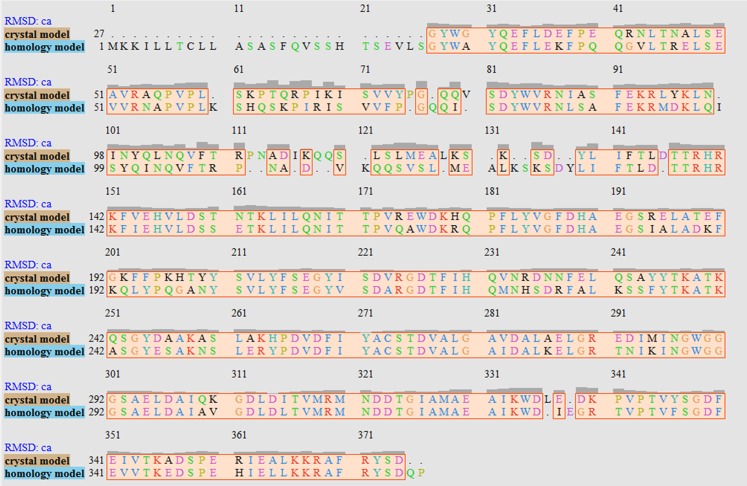
Sequence alignment after superimposition of crystal and homology model shows the RMSD of cα higher in loops, helices and beta-sheets as graphic presentation in Fig. 1c.

The *V. vulnificus* LuxP protein sequence was submitted to the I-TASSER online server for homology structure prediction and five top final models with *C*-scores ranging from −0.22 to −3.16 were generated. Higher *C*-score values correlate with higher confidence levels in the 3D model, and, the model with *C*-score −0.22 was selected for subsequent docking and MD simulation. The top-ranked template identified by LOMETS with the highest normalized *Z*-score of 6.94 is the crystal structure of *V. harveyi* LuxP (PDB: 1JX6), which is one of the closest species to *V. vulnificus*. The surface electrostatic potentials of both protein models were assessed, and an open binding pocket in the model was identified (Figs. 1A and 1B). The 3D model proposed the open conformation (apo structure) as depicted in Fig. 1B represent the structure in the absence of ligand. Protein sequence alignment of the model with template protein identifies the potential helices and beta-sheets that might involve in the major conformational changes upon ligand binding (Fig. 2). Both protein models were docked with fatty acids and the reference compounds to analyze the stability of the complexes via MD simulations.

### Docking and binding pose analysis of fatty acids and reference compounds

**Figure 3:**
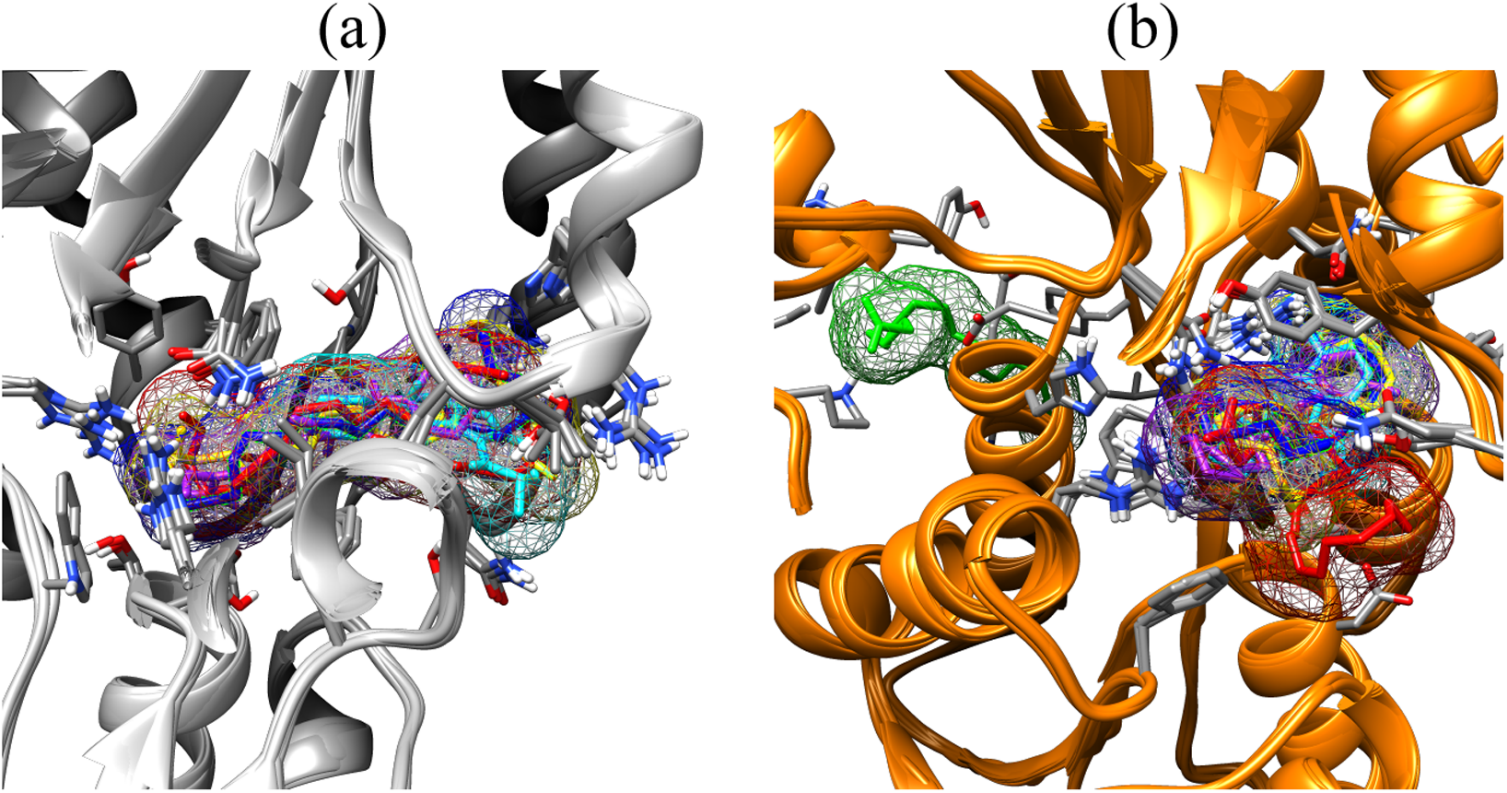
Binding poses of the small molecule metabolites/reference compounds in crystal (A) and homology model (B). Molecule surfaces shown in mesh (50% transparency) fit in the LuxP binding pocket and the interacting residues in LuxP protein were colored by element. Grey chain: crystal model; orange chain: homology model; small molecule metabolites/reference compounds: •4R8 •EPA •LAX •LAX#2 •C19 •C31.

**Figure 4:**
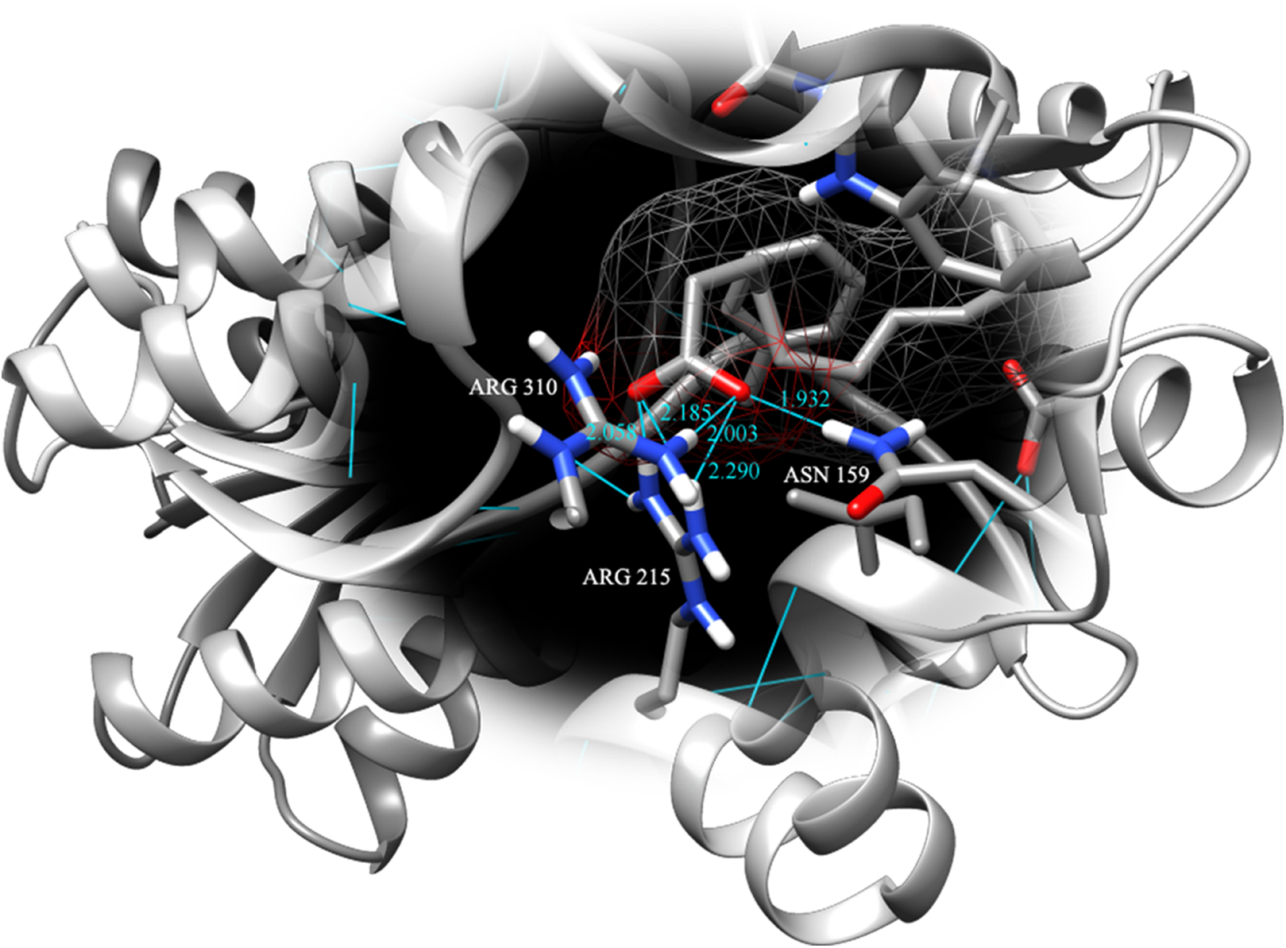
Binding pose of molecule EPA in LuxP crystal model. EPA molecule has been shown in stick format and molecule surface shown in mesh (50% transparency). Residues involved in hydrogen bonding have been labelled and hydrogen bonds were presented in cyan lines; bond length has been shown in unit Angstrom.

Fatty acids that were found to be differentially abundant in *E. fuscoguttatus* that demonstrated their tolerance to the experimental infection by *V. vulnificus* were docked in both *vh*LuxP and *vv*LuxP models using AutoDockVina 1.1.2. The results showed that 4-oxodocosahexaenoic acid (4R8), 5,8,11,14,17-icosapentaenoic acid (EPA), and eicosa-8,11,14-trienoic acid (LAX) have significant binding affinity toward both *vh*LuxP and *vv*LuxP model. The 2D conformation of the fatty acids and the reference compounds with their binding affinity scores are given in Table 1. In the *vv*LuxP model, LAX was found to present a second binding pose (LAX#2) posterior to the *vv*LuxP receptor binding pocket, as shown in Fig. 3, with binding affinity score −5.5 kcal/mol (Table 1). This binding pose of LAX#2 was included in the MD simulation to evaluate the stability of the complex. A significant cut-off binding affinity score of −5.5 kcal/mol was selected; complexes with not significant higher binding affinity scores were excluded from further molecular dynamic simulations analysis. Only parallel models of *vh*LuxP were selected to serve as control models. Reference compounds showed binding affinity score ranging from −7.5 to −8.0 kcal/mol against both LuxP models. eicosapentaenoic acid (EPA) presented the highest affinity toward both LuxP models, with affinity scores of −7.8 and −6.0 kcal/mol. To visualize the interaction pattern, the UCSF Chimera molecular visualization tool was used to generate graphical representations of the hydrogen bonds (H_bonds) formed between the amino acid residues. Details of the hydrogen bonds formed between the amino acid residues with the fatty acids were reported in Table 2. The EPA molecule was found to interact with three key binding residues in *vh*LuxP receptor protein, as shown in Fig. 4. All these complexes were further assessed by MD simulation for structural behavior and flexibility analysis.

**Table 1:** 2D structures of the fatty acids and reference compounds 2D structures of the fatty acids and reference compounds used in molecular docking, with the binding affinity score toward *vh*LuxP/*vv*LuxP computed using AutoDockVina1.1.2.

	Name	2-D structures	Affinity score (kcal/mol)	
			*vh*LuxP	*vv*LuxP
Fatty acids	4-Oxodocosahexaenoic acid (4R8)	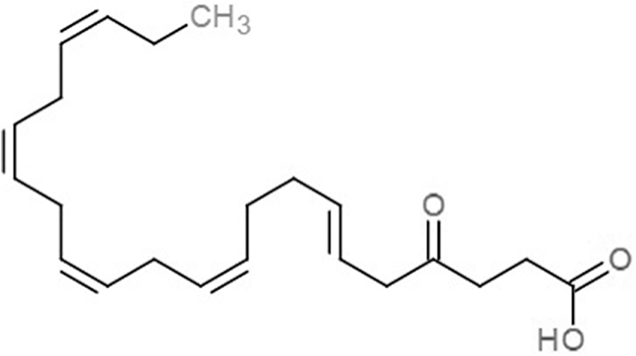	−7.6	−6.0
	5,8,11,14,17-Icosapentaenoic acid (EPA)	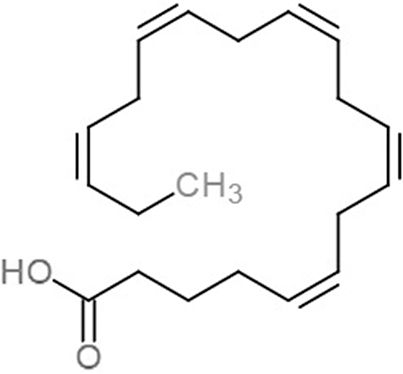	−7.8	−6.0
	Eicosa-8,11,14-trienoic acid (LAX)	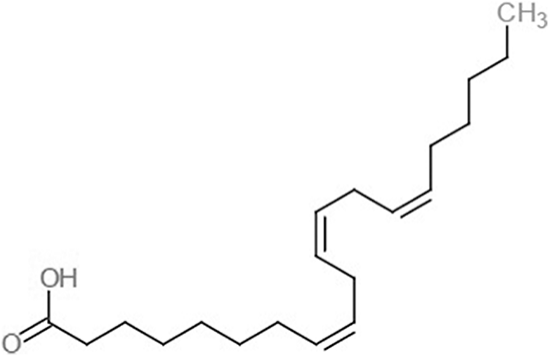	−7.4	−5.9 −5.5
	Icosanoic acid (DCR)	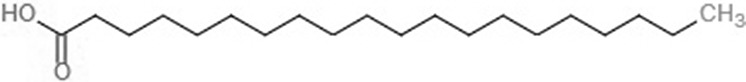	−6.6	−4.4
	Linoleic acid (EIC)	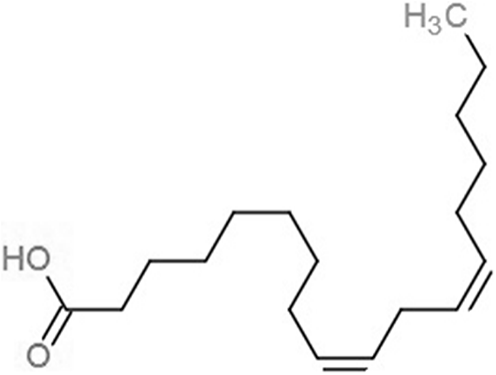	−7.2	−4.8
	9-Octadecenoic acid (ELA)	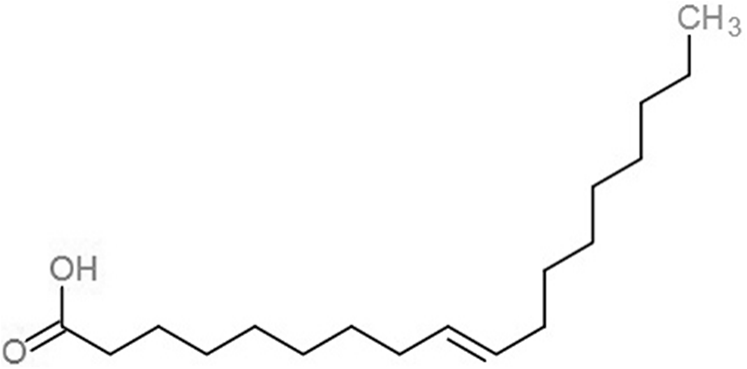	−6.8	−4.6
	Alpha-linolenic acid (LNL)	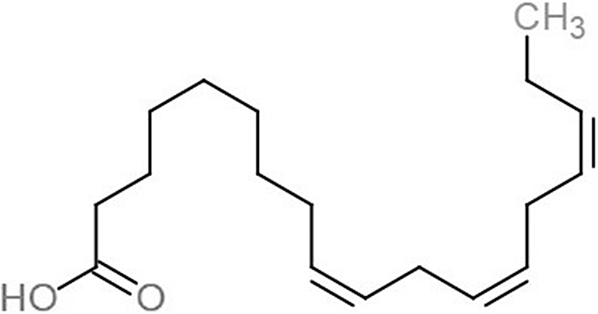	−7.1	−4.9
	Oleic acid (OLI)	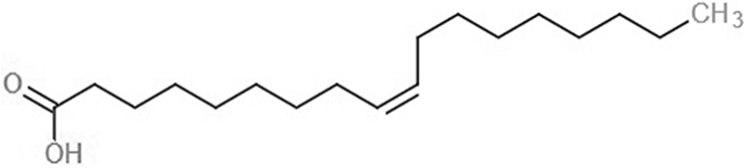	−6.9	−4.9
	Palmitoleic acid (PAM)	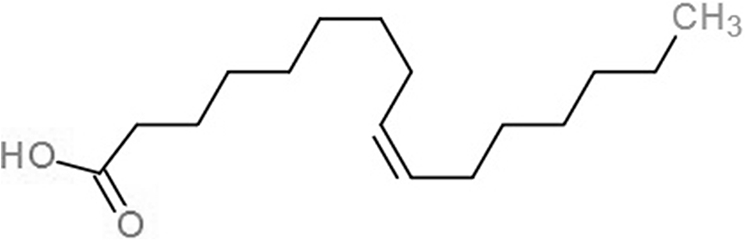	−6.7	−5.4
Commercial compounds	Compound 19 (Zhu et al., 2012)	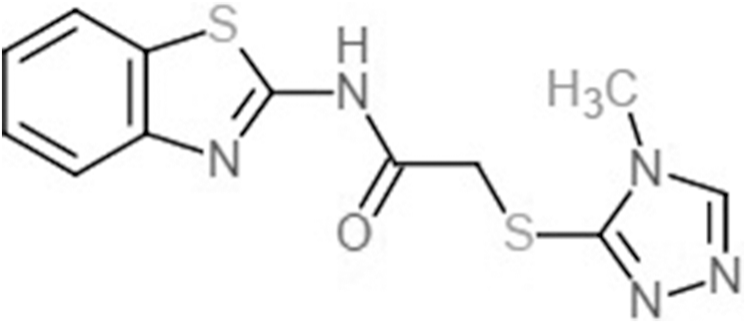	−8.0	−7.5
	Compound 31 (Zhu et al., 2012)	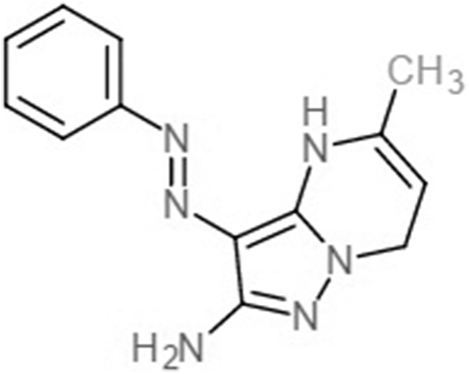	−7.6	−7.6

**Table 2:** H_bond interactions between fatty acids/reference compounds and key residues in the LuxP binding pocket.

	Fatty acids/compounds	Hydrogen donor::Hydrogen acceptor (H_bond distance, Å)
*vh*LuxP	4R8	GLN 116.A NE2 4R8 365.B O3 (2.009); ASP 136.A N::4R8 365.B O1 (1.863); THR 137.A N::4R8 365.B O2 (1.650); THR 137.A OG1::4R8 365.B O2 (1.754)
	EPA	ASN 159.A ND2::EPA 365.B OB (1.932); ARG 215.A NH1::EPA 365.B OA (2.058); ARG 215.A NH2::EPA 365.B OA (2.185); ARG 215.A NH2::EPA 365.B OB (2.290); ARG 310.A NH1::EPA 365.B OB (2.003)
	LAX	SER 79.A OG::LAX 365.B O1 (1.919); ARG 215.A NH1::LAX 365.B O2 (2.295); ARG 215.A NH2::LAX 365.B O2 (2.131); ARG 310.A NH1::LAX 365.B O2 (1.878); ARG 310.A NH2::LAX 365.B O1 (2.118); ARG 310.A NH2::LAX 365.B O2 (2.688)
	C19	–
	C31	GLN 116.A NE2::C31 365.B NAM (2.016)
*vv*LuxP	4R8	ASN 159.A ND2::4R8 367.B O2 (1.836); ARG 215.A NH1::4R8 367.B O3 (2.319); ARG 215.A NH2::4R8 367.B O3 (1.728)
	EPA	ARG 310.A NE::EPA 367.B OA (1.947); ARG 310.A NH1::EPA 367.B OA (2.239)
	LAX	ARG 310.A NE::LAX 367.B O2 (1.732); ARG 310.A NH1::LAX 367.B O2 (2.688)
	LAX#2	LYS 3.A NZ::LAX 367.B O2 (2.040); GLN 32.A N::LAX 367.B O1 (1.986)
	C19	–
	C31	C31 367.B NAL::THR 266.A OG1 (2.199); C31 367.B NAL::THR 266.A OG1 (2.199)

### Molecular dynamic simulations

**Figure 5:**
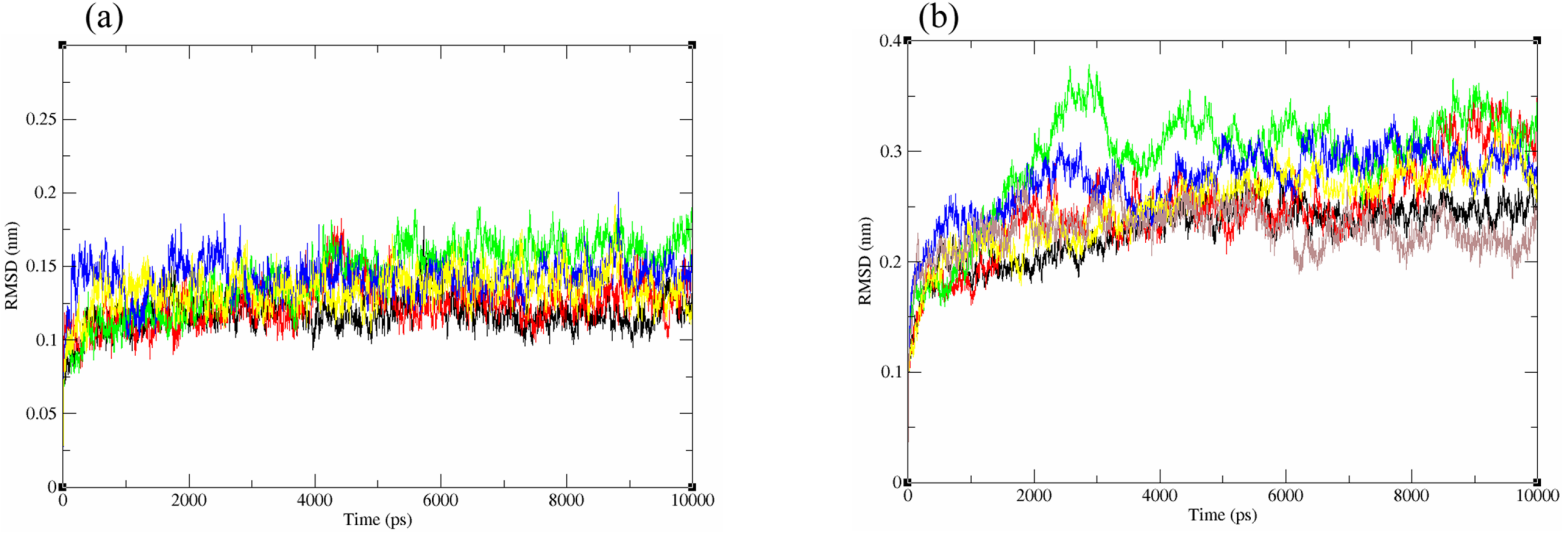
Backbone RMSD of crystal (A) and homology model (B) have been shown in figure against the initial structure during 10 ns MD simulation. Crystal model: •4R8 •EPA •LAX •C19 •C31 Homology model: •4R8 •EPA •LAX •LAX#2 •C19 •C31.

**Figure 6:**
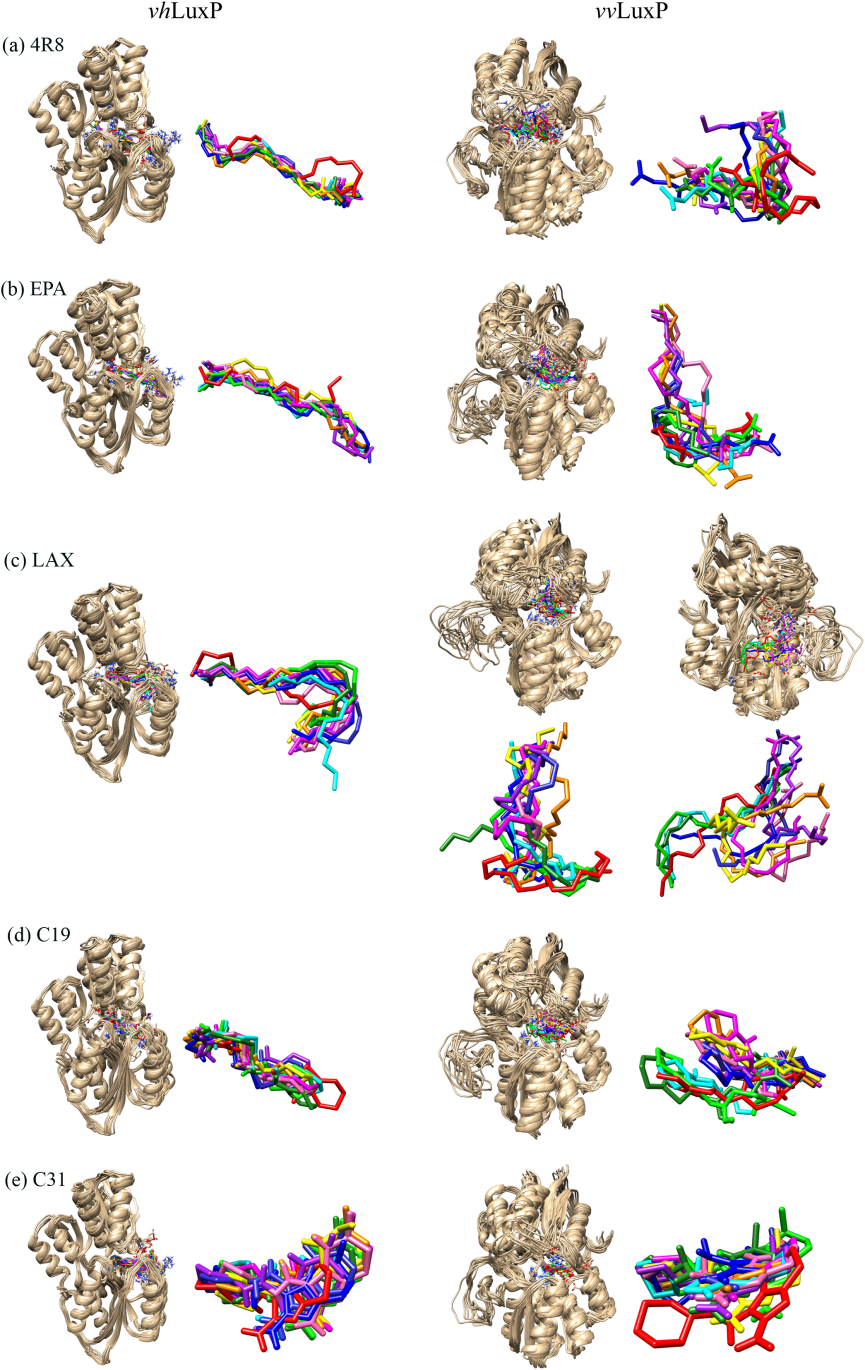
Superimposition of trajectory structures at 1 ns interval of 10 ns MD simulation. Proteins presented as grey colored ribbon format. Snapshot of the superimposed small molecule ligands at 1 ns interval in the LuxP ligand binding site (shown in stick format). Color coded for different time frame extracted from trajectory structure. (A) 4R8, (B) EPA, (C) LAX, (D) C19, (E) C31.

**Figure 7:**
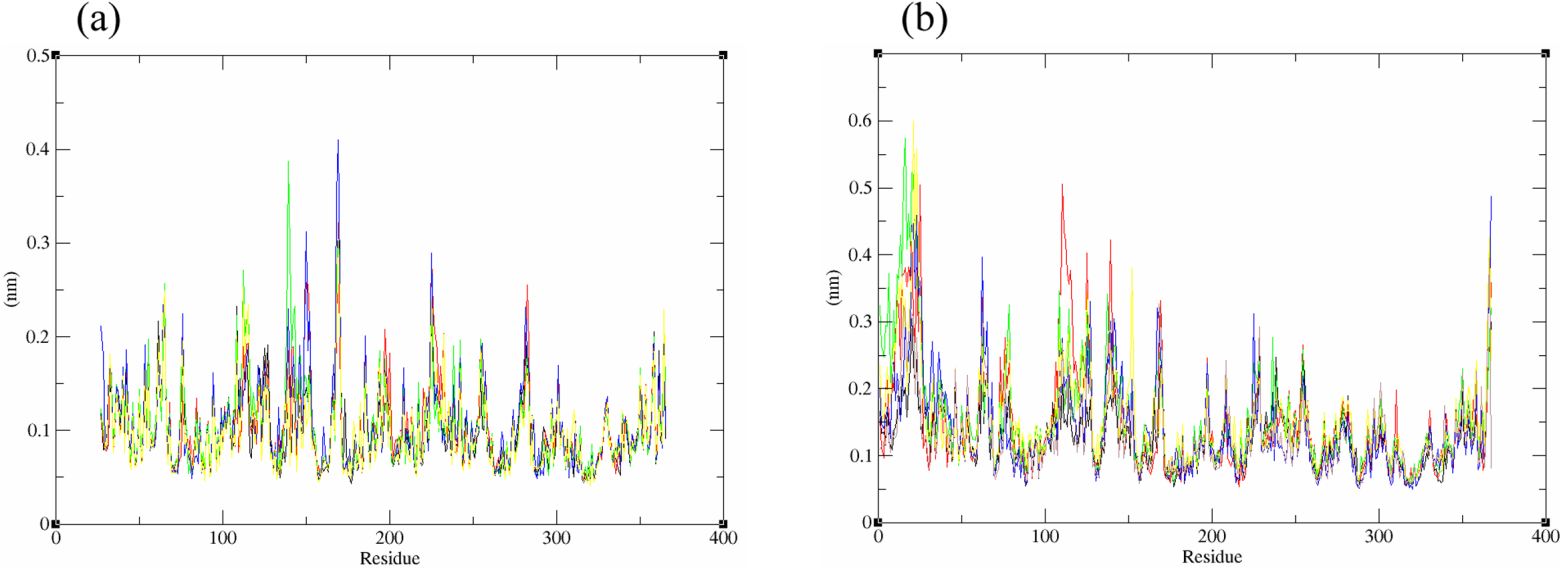
Residue RMSF of the protein-ligand complexes from both the crystal (A) and homology model (B) generated during the trajectory period of 10 ns MD simulation. Crystal model: •4R8 •EPA •LAX •C19 •C31 Homology model: •4R8 •EPA •LAX •LAX#2 •C19 •C31.

The structural behavior and flexibility of *vh*LuxP and *vv*LuxP docked with fatty acids and reference compounds were assessed by 10 ns of MD simulation using Gromacs 4.6.5 for each complex. Preliminary simulation for 50 ns was assessed and the stabilized protein backbone was identified after 10 ns of simulation. Due to the limitation in computing power and the preliminary simulation result, the structural behavior and flexibility of LuxP complexes were assessed for 10 ns of MD simulation. The root mean square deviation values of both *vh*LuxP and *vv*LuxP were calculated against the initial structure in the protein–ligand complexes and plotted using the 3-D plotting tool Xmgrace to compare the protein backbone stability. The backbones of the *vv*LuxP–ligand complexes showed significant fluctuation compared to the *vh*LuxP–ligand complexes (Fig. 5), implying that the binding of fatty acids and the reference compounds in *vh*LuxP is more stable and does not affect the protein backbone stability. The LuxP–ligand complexes were snapshot at one ns intervals from the 10 ns MD production trajectory and were superimposed to assess the ligand binding stability as in Fig. 6, observed the stability of the ligand binding position in each of the complexes. Meanwhile, the stability of the LuxP–ligand complexes was examined by calculating the residual mobility. The Root Mean Square Fluctuation of the trajectory from the MD simulation for each complex was calculated, and the protein residual fluctuations in LuxP–ligand complexes are minimal for both LuxP models (Fig. 7).

### Hydrogen bond analysis

**Figure 8:**
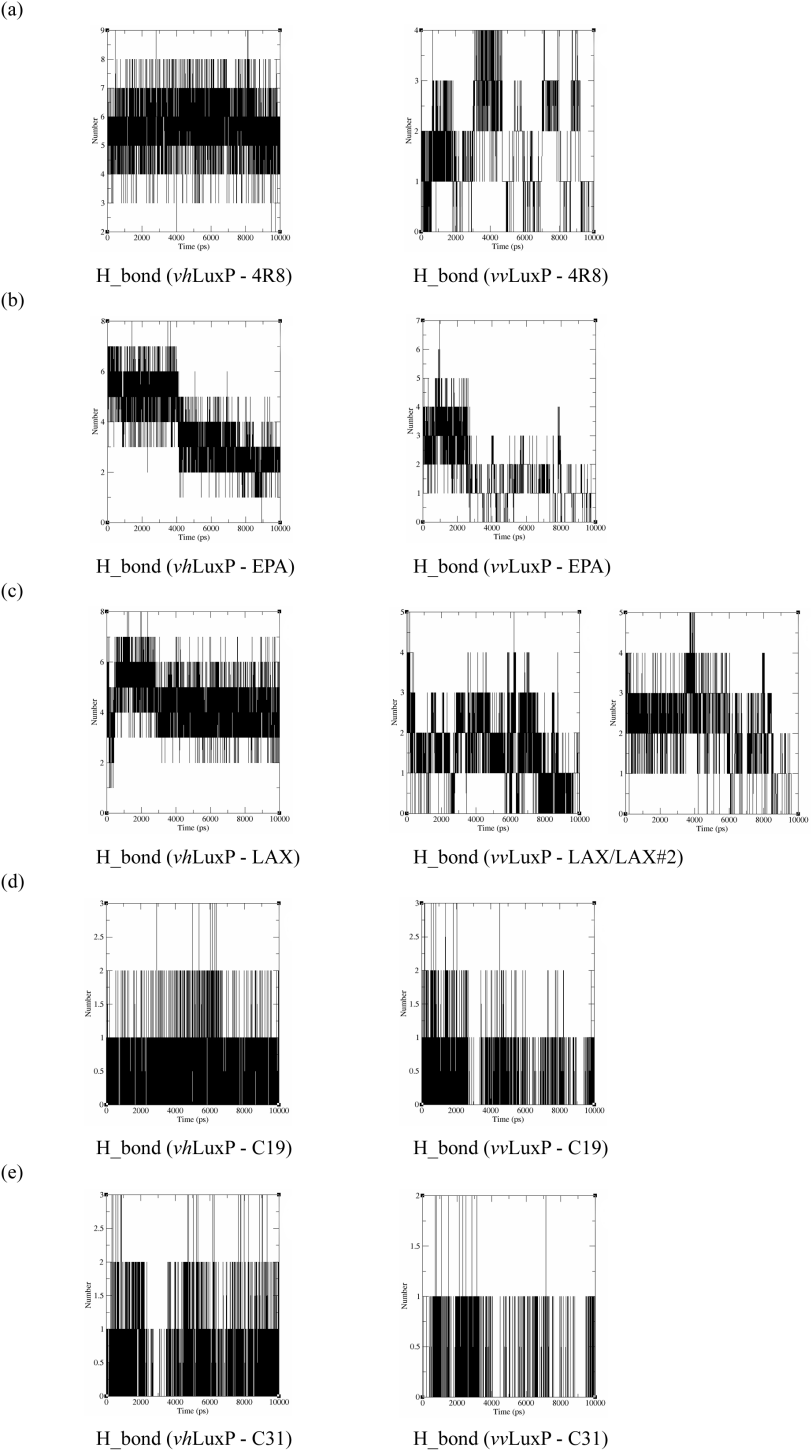
Total number of hydrogen bond interactions between LuxP (crystal and homology model) and small molecule metabolites/reference compounds (A) 4R8; (B) EPA; (C) LAX; (D) C19; (E) C31.

The binding stabilities of the fatty acids/reference compounds in both LuxP models were monitored during the trajectory period of the MD simulations. The stabilities of the LuxP–ligand complexes were evaluated by calculating the H_bond profiles using the g_hbond tool of Gromacs (Van der Spoel et al., 2013; Hess et al., 2008). The analysis revealed that the protein–ligand complex of 4-oxodocosahexaenoic acid (4R8) in *vh*LuxP has the highest (5.676) average number of hydrogen bonds per timeframe during the MD simulation period (Fig. 8). The average numbers of H_bonds observed in EPA and eicosa-8,11,14-trienoic acid (LAX) in *vh*LuxP were 3.776 and 4.331, respectively. Poor H_bond interaction was observed in *vv*LuxP, with 1-2 H_bonds on average throughout the MD simulation (4R8: 1.732 H_bonds; EPA: 1.615 H_bonds; LAX: 1.482 H_bonds; LAX#2: 1.958 H_bonds). However, the least H_bond interaction was recorded in both reference compounds in *vh*LuxP and *vv*LuxP (*vh*LuxP::C19 0.459 H_bonds, *vh*LuxP::C31 0.404 H_bonds; *vv*LuxP::C19 0.238 H_bonds, *vv*LuxP::C31 0.112 H_bonds) (Fig. 8).

### Re-scoring of interaction and binding free energies

The complex stability is further assessed by calculating the binding free energy using the g_mmpbsa tool (Kumari et al., 2014). Polar and non-polar energy terms were calculated for each complex. Both *vh*LuxP and *vv*LuxP hindered the binding of all fatty acids and reference compounds in terms of the polar solvation energy, which was recorded between 108.944 (lowest polar solvation energy) and 494.049 kJ/mol (highest polar salvation energy) (Table 3). Various non-polar energy terms (Van der Waals, VdW; SASA; and SAV) are favorable for all fatty acids and the reference compounds that bind in both LuxP models. Although both reference compounds have the least hydrogen bond interaction in *vh*LuxP and *vv*LuxP (Fig. 8), Compound C19 has the lowest binding free energy recorded, which was mainly contributed by low electrostatic energy in both *vh*LuxP (496.516 kJ/mol) and *vv*LuxP (−336.767 kJ/mol)and stabilized the binding of C19 in LuxP.

**Table 3:** Interaction energy and binding free energy of *vh*LuxP–ligand and *vv*LuxP–ligand complexes calculated using the MM-PBSA approach.

protein	Fatty acids/compounds	Electrostatic energy (kJ/mol)	Polar solvation energy (kJ/mol)	Van der Waal energy (kJ/mol)	SASA energy (kJ/mol)	SAV energy (kJ/mol)	WCA energy (kJ/mol)	Binding energy (kJ/mol)
*vh*LuxP	4R8	−164.351 ± 38.398	476.780 ± 25.417	−184.121 ± 13.696	−22.966 ± 0.892	−173.708 ± 14.654	77.343 ± 17.396	8.977 ± 28.950
	EPA	15.865 ± 43.571	363.125 ± 32.139	−143.807 ± 14.356	−21.987 ± 1.102	−143.758 ± 19.078	69.899 ± 15.702	139.337 ± 40.764
	LAX	−2.922 ± 28.589	358.883 ± 21.159	−151.554 ± 15.144	−20.554 ± 1.107	−135.507 ± 17.623	69.548 ± 15.643	117.894 ± 31.620
	C19	−496.516 ± 38.609	494.049 ± 27.222	−137.836 ± 13.859	−16.142 ± 0.523	−124.166 ± 10.287	56.472 ± 12.644	−224.139 ± 20.989
	C31	−15.257 ± 5.854	124.379 ± 14.281	−111.304 ± 13.488	−14.726 ± 0.632	−85.076 ± 12.324	46.959 ± 10.506	-55.024 ± 17.178
*vv*LuxP	4R8	−38.303 ± 27.662	219.796 ± 42.493	−140.233 ± 16.467	−19.564 ± 1.624	−121.480 ± 15.673	70.640 ± 16.114	−29.144 ± 25.596
	EPA	34.517 ± 44.631	189.767 ± 78.739	−126.031 ± 11.995	−18.294 ± 1.173	−112.833 ± 14.868	64.612 ± 14.851	31.739 ± 44.926
	LAX	−38.705 ± 56.060	279.238 ± 51.851	−152.987 ± 16.150	−19.424 ± 1.058	−124.314 ± 14.943	68.580 ± 15.511	12.389 ± 36.408
	LAX#2	−3.545 ± 82.939	240.374 ± 116.852	−91.236 ± 25.199	−13.479 ± 2.817	−75.400 ± 25.372	69.699 ± 16.152	126.413 ± 44.006
	C19	−336.767 ± 42.665	399.581 ± 50.967	−115.842 ± 14.969	−14.650 ± 0.966	−91.855 ± 11.493	57.774 ± 12.937	−101.759 ± 26.247
	C31	−16.970 ± 4.250	108.944 ± 9.451	−121.784 ± 10.345	−14.950 ± 0.619	−102.954 ± 7.247	46.288 ± 10.356	−101.427 ± 11.162

**Notes:**

Each value represents the average value calculated from 20 snapshots at 0.5 ns intervals of the 10 ns MD production run.

SASA, solvent accessible surface area; SAV, solvent accessible volume; WCA, Weeks–Chandler–Andersen.

## Discussion

It has been reported that LuxP undergoes conformational changes upon (AI-2) binding to form the AI-2-LuxPQ complex (Stock, 2006; Zhu et al., 2012). Template-model based alignment (Fig. 2) has revealed the potential helices and beta-sheets that might be involved in the major conformational changes upon ligand binding, as shown in Fig. 1C. Crystal structure of LuxP without endogenous ligand is not available due to the fact that in order to crystallize the target protein, the compositional and conformational stability of the protein are prerequisite (Deller, Kong & Rupp, 2016). The binding of endogenous ligand, furanosyl-borate diester (AI-2) as observed in *vh*LuxP (crystal structure) involving the H_bond interactions with three key residues, which are ASN159, ARG215, and ARG310. Although the binding affinity score of LAX was lower than the other molecules, it is still considered as a potential candidate to interact with LuxP because it forms H_bond with the key residue of ARG310 in *vv*LuxP. It also has the highest number of H_bonds with the most key residues in the binding pocket of *vh*LuxP (Table 2). Surprisingly, reference compound 19 shows no H_bond interaction in the binding pocket despite having been proven to inhibit AI-2 quorum sensing (Zhu et al., 2012). The structural behavior and flexibility of *vh*LuxP and *vv*LuxP were assessed and the results were not as anticipated as the protein backbone of *vv*LuxP is expected to be more flexible since it is known to undergo major conformational changes upon ligand binding (Stock, 2006; Zhu et al., 2012). This may be due to the stability of ligands that bind inside the binding pocket that is crucial to determine the efficiency of the ligand in inhibiting LuxP protein. However, the relatively short MD simulation of 10 ns could be another limiting factor to study and conclude the protein flexibility. Snapshots on the LuxP–ligand complexes were taken at one ns intervals from the 10 ns MD production trajectory and were superimposed to assess the ligand binding stability (Fig. 6). It showed that the positions of the ligands bound in *vh*LuxP are more confined and stable compared to *vv*LuxP. The open binding pocket of *vv*LuxP possesses higher accessible volume, and therefore the interaction of the ligand inside the binding pocket must be stronger and more specific in order to stabilize. When the stability of the LuxP–ligand complexes was examined, no significant fluctuation on the residues involved in the interaction was recorded in both *vv*LuxP and *vh*LuxP model. The simulation results showed similar dynamics for *vv*LuxP model and the control *vh*LuxP model, further strengthening the proposed open conformation of LuxP which was depicted in Fig. 1B to represent the LuxP apo structure. Although the average number of H_bonds varies significantly among the different complexes, the binding of these molecules in the protein binding site is relatively stable, except for the *vv*LuxP–LAX#2 complex, in which a higher level of fluctuation in the ligand binding pose is observable (Fig. 6). Nevertheless, less H_bond interaction is observed in both reference compounds, implying that the binding stability is contributed by the electrostatic forces. The continuous contribution of H_bond interactions in the binding pose analysis suggests that these fatty acids possess potential stable interaction with the LuxP protein.

The binding free energies calculated using the MMPBSA approach indicated that among the three fatty acids, 4R8 displayed the lowest binding free energy (−29.144 kJ/mol) in *vv*LuxP. The lower binding free energy suggests that the *vv*LuxP-4R8 complex is more stable than the *vv*LuxP–EPA and *vv*LuxP–LAX complexes, which have higher binding free energies of 31.739 and 12.389 kJ/mol, respectively. The results obtained from this study suggest that 4R8 is a potential candidate possesses molecular interaction with quorum sensing receptor LuxP protein. Nevertheless, further in vitro/in vivo experiments are recommended to evaluate the biological effect of 4R8 interaction with the quorum sensing receptor LuxP of quorum sensing signaling in *Vibrio*.

## Conclusions

The MD of the LuxP–ligand complexes, as shown by MD simulation, identified 4-oxodocosahexaenoic acid (4R8) as a potential candidate to interact with the LuxP receptor protein. The binding of 4R8 in both *vh*LuxP and *vv*LuxP displayed a stable protein backbone conformation during the MD simulation and a stable ligand binding position with the highest hydrogen bond interactions within the 10 ns MD simulation. The computational analysis results suggest a molecular interaction of the fatty acid 4R8 with the quorum sensing receptor LuxP. This knowledge is highly valuable for further in vivo/in vitro analysis to evaluate the effect of this molecular interaction in the biological signaling of *Vibrio* quorum sensing system.

## Additional Information and Declarations

### Data Availability

The following information was supplied regarding data availability: The raw data are available at the NCBI Protein Databank, accession number WP_011152474.1.
